# Exploring patient referral practices of traditional healthcare practitioners in Soshanguve, Gauteng Province, South Africa

**DOI:** 10.4314/ahs.v24i1.31

**Published:** 2024-03

**Authors:** Eugene Musiiwa Makhavhu, Mmajapi Elizabeth Masala-Chokwe, Tendani Sara Ramukumba

**Affiliations:** 1 Nursing Science Department, Sefako Makgatho Health Sciences University, Pretoria, South Africa; 2 Adelaide Tambo School of Nursing Sciences, Tshwane University of Technology, Pretoria, South Africa

**Keywords:** referral system, patient referral, traditional healthcare, allopathic healthcare

## Abstract

**Introduction:**

It may be necessary for healthcare professionals to refer patients to other professionals and institutions that are able to provide the care needed by patients, depending on the severity each case presents, healthcare requirements, and resources available to offer care. In healthcare generally, patient referral is standard procedure; however, in South Africa, referral patterns between allopathic and traditional healthcare practitioners are unknown, and this is a problem yet to be addressed.

**Objectives:**

The study objective was to explore patient referral practices from the perspectives of traditional healthcare practitioners of South Africa.

**Methods:**

A qualitative, exploratory and descriptive design was employed to collect data from 11 traditional healthcare practitioners who were sampled using snowball sampling. An individual semi structured interview schedule was used to collect data. Content analysis was used to analyze the data.

**Results:**

Patient referral was common practice in traditional health practices, however not reciprocal between the traditional and allopathic healthcare. Several indications for patient referral to allopathic healthcare practitioners included management of chronic conditions amongst others.

**Discussion:**

Patient referral being an important part of healthcare practices should be promoted and regulation and traditional health practices improved to promote safe referral practices and curb unsafe self-referrals by patients.

## Introduction

Patient referral is a written request to take over treatment of a patient or to provide input into patient management that typically entails contact between the referring practitioner and the receiving practitioner^1^. It is a form of care taken from one healthcare professional to another professional or to another health service for further diagnosis and treatment.

The practice of patient referral is an integral part of healthcare in general and it can positively affect patient health outcomes if done correctly. It is a typical component of patient care, with referrals being used in roughly 5% of cases treated in primary care^2^. An effective referral system is essential to save lives and to promote continuity and quality healthcare^3^. A well-functioning referral system is central to an effective healthcare delivery system and achievement of universal health coverage^4^. This is what is urged in the National Health Insurance bill of South Africa, in line with section 27 of the constitution which seeks to ensure that everyone has access to high-quality healthcare services in the Republic^5^. Part of the referral process is also aimed at coordinating healthcare to mitigate dealing with large numbers of patients in one facility when others can be seen at a lower level of care.

In the South African context, the allopathic healthcare system includes what is usually termed biomedical healthcare or modern medicine which is offered in healthcare facilities that are both public and private, at various levels including clinics and hospitals. These levels of care differ depending on patient's health status and their healthcare needs at the time. The referral system is hierarchal and is based on principles of primary health care. These includes equity in healthcare service delivery, affordable and appropriate service accessibility, people empowerment, and sustainable service provision.^6^ In the public sector, patients would present themselves at a Primary Health Care clinic as their first formal point of contact4 and depending on their condition and need for referral, they may be referred to the next level of care. Example of this is when a patient is referred from the clinic to a district hospital. Should management be unsuccessful at a district hospital or specialized care is needed, next referral point is a tertiary or academic hospital. Worldwide, the major types of healthcare facilities include Primary healthcare and the hospital, and this is the case in most countries^7^. However, South Africa is different in that there is another unconventional method of traditional healthcare practices where patient referrals could also be discussed.

The South African Traditional Health Practitioners' act 22 of 2007 defines traditional health practices as the performance of a function, activity, process, or service based on a traditional philosophy that includes the utilization of traditional medicines or practices^8^. Traditional health practitioners in South Africa can be categorized as diviners, popularly known in local communities as Sangomas, herbalists, spiritual healers, and traditional birth attendants^9^.

An estimated 80% of black South African consult a traditional health practitioner before a hospital or clinic^10^. People would consult with any category of practitioner, however, health-seeking behavior in South Africa can at times be parallel where people consult a traditional health practitioner and an allopathic healthcare facility. Although their practices are not formalized by means of government regulation, traditional health practitioners in South Africa are well known and community members often go to consult for healthcare needs believed to be better solved by them. In efforts to formalize and regulate traditional practices, integrating them with allopathic healthcare systems has been a discussion point in different forums and also indicate patient referrals amongst practitioners.

Traditional health practitioners prefer co-referrals between the two healthcare systems to exist and facilitate integration^11^, which will see the allopathic and traditional healthcare practices co-exist. Such referral does not exist currently, and this may be linked to poor regulation of traditional health practices. Integration could likely improve patient referral and also bring about an opportunity to see cross-referral of patients and exchange of knowledge and experience^12^.

While patient referral is common practice in allopathic healthcare systems, the same cannot be confirmed for traditional health practice and the referral practices of traditional health practitioners in South Africa are not known, neither are they widely documented.

## Methods

### Study design and setting

A qualitative^13^ exploratory and descriptive design was used to explore patient referral practices from the perspectives of traditional health practitioners. The study was conducted in a township about 35 kilometers north of the City of Tshwane in Gauteng Province, South Africa. The community has a substantial existence of traditional healthcare practitioners who would usually be noticed by their attire and beads around their wrists and ankles. Sometimes they are seen when performing their rituals and drumming better known in the townships as *mantshomane*. This township with a population of approximately 878 960 people^14^ is a low to middle-income community. Access to allopathic healthcare is limited to one community health center, and seven public clinics. There is no government hospital in the community, however, there is one private hospital for people who can afford the services.

### Sampling

Snowball sampling^15^ was used to sample traditional healthcare practitioners. Although there is a sizeable number of practitioners, their practices are deemed sacred, as a result the sampling method appropriate for this study was snowball sampling. The researcher contacted a known herbalist who contacted another traditional healthcare practitioner, and snowball materialized until the ninth participant when no new information emerged. Two more participants were interviewed to confirm all findings and saturate the data. A total sample of 11 traditional healthcare practitioners who were active in practice took part in this study. All of the participants were over the age of 18, were fluent in Setswana, which is widely spoken in the area, and some could also speak English.

### Data collection

Individual face to face interviews were conducted by means of a semi-structured interview guide. The interview guide was drafted in English. A translation to Setswana was done by the primary investigator during data gathering. For those individuals who could grasp the language and respond appropriately, English was also used. An audio recorder was used and participants were made aware of the recording. Informed written consent was obtained from participants prior to data collection.

### Data analysis

All recorded interviews were listened to and transcribed verbatim by the primary investigator. Content analysis^16^ was used to analyze the data. After transcription, each interview was read, reviewed and compared to the next to extract commonalities in participants' responses and conclude on common themes. Analysis was done by the authors of this paper and an independent coder was used to confirm the themes identified. Four themes emerged from the analysis.

### Trustworthiness

Trustworthiness was ensured by confirming collected data and accurate interpretation done by all authors of the paper, furthermore, an independent coder confirmed the data and themes extracted. For dependability, the criterion for participation was clearly laid out to ensure that sampled participants were able to respond adequately to the research question to satisfy the objective of the study. For credibility, the study received ethical clearance from the higher education institution.

### Ethical consideration

Prior to data collection, ethical approval was sought from the institution's ethics committee. Participation was voluntary and participants were able to provide the investigator with signed informed consent after reading an information leaflet with the researcher. Principles of nursing research, beneficence and non-maleficence were adhered to throughout the study process and this was ensured by securing participants' well-being and protecting them from discomfort and harm^15^. To maintain anonymity, participants were not identified during the data collection procedure. To protect the participants' privacy and confidentiality, each interview was performed privately in a separate room. Participants were assured of their rghts to self-determination, including the ability to leave the study if they felt uncomfortable, and codes were used to identify data at transcribing.

## Results

### Socio-demographics

The study participants comprised 11 participants, nine female and two male traditional health practitioners. Of the 11 participants, 10 were practicing as Sangomas/diviners and one practicing as a herbalist. Of the 10 Sangomas, two were in dual practice as both Sangoma and spiritual healer. Participants were aged between 26 and 58 years and had been practicing in their respective fields for periods between four years and others longer than 30 years. Four of the participants were employed in formal jobs while the others were only practicing as traditional health practitioners.

### Past and likelihood for future referral of patient to allopathic healthcare practitioners

This study found that participants in their majority had previously referred clients to allopathic healthcare providers. Of the 11 participants, eight indicated that they have referred some of their patients to allopathic healthcare practitioners in the past while the other three had never referred. All participants who took part in this study indicated that they would potentially refer patients to allopathic healthcare practitioners should the need arise in future. Below are some quotations from participants' responses.

*Yes, I have referred before. And I would say probably more than ten people. Especially in children and if I see that the child is sick, I tell them [the parents to] go to a hospital.”* **[THP1, Female, 46 years old]**

*“I have referred a patient to a clinic before and I mostly accompany them. Especially to a clinic where I have some relations with the nurses.” [* **THP2, female, 57 years old]**

*“Yes, I would definitely refer. For example, these illnesses are not the same. If you come and I know you are HIV positive, I would definitely say that you go to the hospital”* **[THP8, female, 28 years old].**

*“You see, we need to accept when we have met a challenge in terms of our treatment, and thus look for other options. So, if my patients are really looking sick and I am unable to help them, I will definitely refer them to the nearest doctor who can better treat them.”* **[ITHP10, Male, 47 years old]**

### Referral of patients from allopathic healthcare practitioner

Although traditional healthcare practitioners refer patients to allopathic healthcare practitioners, because they did not obtain referrals from allopathic healthcare providers, it was discovered in this study that referrals are not reciprocated. Only one participant had previously received an informal referral from an allopathic healthcare practitioner known to them at a personal level. Some participants cited seeing patients based on self-referrals. Quotes below indicates some of the participants' responses.

*“No, I haven't received patients from them [allopathic healthcare], but a patient can come to me and tell me that they have been to a hospital and it didn't work and I see them”* **[THP1, Female, 46 years old]**

*“No, I haven't [received a referral]. But a lot of people do come and they say they have been to a clinic or a particular hospital but it's [the treatment] not working”* **[THP4, Female, 26 years old]**

*“A lot of times, they come themselves and state that they have been to a clinic or hospital and didn't get help. So, I help them”* **[THP5, female, 36]**

*“Not as a referral from the Dr or nurse, but the clients do say that they have been going to the clinic or hospital several times and do not get the required assistance”* **[THP8, female, 28 years old].**

*“Yes, they do [refer patients]. The clinic nearby does. I remember they once called us in this clinic when there was a different clinic manager. But you need to also remember that nurses are different as well. So, the former clinic manager was very understanding and we use to work very well with her, but with the new one, it's a bit difficult.”* **[THP2, female, 57 years old]**

### Indications for patient referral to an allopathic healthcare practitioner

Participants mostly cited patients who were known to have complications from chronic conditions such as diabetes and hypertension to be on their list of patients to refer to allopathic healthcare practitioners. Patients would be on treatment from their clinic or hospital and to prevent complicating treatments, they encourage them to visit a doctor or nurse prior to consultation with them. Other indications included clients who looked lethargic and weak for unknown reasons. Some participants further mentioned that if there is a known contraindication to the treatment they would prescribe for the patient, they are likely to refer. Patients who are suspected to have TB are also referred to be started on treatment. Below are some of the quotes by participants.

*“If there is nothing I can do for the client, then yes, I do refer them. For example, an HIV positive client who is very weak and sick I will definitely refer them to the clinic because there are some herbs that we may not give to such a person, so what do you do. You refer them to the hospital where you know they will get help”* **[THP3, female, 36 years]**

*“Yes, I can refer them. There are those clients who would come and I can see that they are sick spiritually and physically. The bones I throw sometimes do tell me that the person is suffering from a blood disease so I will tell the person to go to a hospital maybe they need blood transfusion and a drip, maybe they are dehydrated. So, I can help them deal with the spiritual issues and encourage them to go to the hospital to get help with the physical being”* **[THP5, female, 36 years old]**

*“Yes, if a patient comes and I see that this is beyond me I will first ask, have you been to the clinic? Have you been checked at the clinic? If not, I will tell them to go to the clinic and come back after they have been assisted. I will tell them that I can only help them after they have been to a clinic. They will check your BP, and other things that I can't throw the bones for and then I can assist after that”* **[THP7, female, 46 years old]**

*“Sometimes they require water [referring to intravenous infusion of fluid] so I send them there and they can come back when they are better so we can continue with our treatment”* **[THP9, female, 39 years old]**

### Method of patient referral

Patient referral was done at each participant's discretion, as a result, the methods of referring patient differed from one participant to another. Verbal referrals were popular while one participant had a referral letter used by the local traditional health practitioners' organization. Another practitioner cited personally escorting her patients to the nearest clinic.

*“I go with them mostly; I get satisfied when I go in with them so I can tell the nurse or doctor receiving the patient as to why I am referring them and what I found so far”* **[THP2, female, 57 years old]**

*“I actually have a referral letter. Our association has one and refers to another traditional practitioner as well. I write all I know including when I started working with the client and state that I am suspecting this and that and request that they must manage further. So yes, I have been doing that.”* **[THP6, female, 58 years old]**

*“I actually prefer to tell my patients to go to the hospital or clinic before we start working. It is hard sometimes if I write them something because the doctors and nurses will not take the referral letter seriously and sometimes the patient will be turned back. So it is best sometimes to just tell them to go and we will continue when the doctors have finished with them”* **[THP10, Male, 47 years old]**

*“But if you refer the patient, they turn them back. So, you are left not knowing what to do. So as for me, I don't refer formally, I just advice my patients to go”*. **[THP 11, Male, 50 years old]**

## Discussion

This study was conducted to explore the referral practices applied by traditional healthcare practitioners in a community in the City of Tshwane, in South Africa. Findings made reference to poor referral practices between allopathic and traditional health practitioners. The undocumented, poor referral practices could lead to poor patient follow-up in cases where a patient is referred from a traditional health practitioner or in cases of patient self-referrals. Poor regulation of traditional healthcare practices may be one reason why there aren't as many patients being referred by allopathic healthcare providers as has been highlighted. Promoting integration of healthcare between the two systems may lead to promotion of safe patient referral.

Such promotion may bring a better understanding of the alternative methods of treatment or the combined healthcare that patients seek. Furthermore, it can assist with better patient management without risking complications. Patients are users of healthcare, and the systems should support them regardless of their health-seeking behavior. Referral of patients which is known and better understood by practitioners of both healthcare systems can be useful in promoting continuity in patient care.

This study did not seek referral practices of allopathic healthcare practitioners to traditional healthcare practitioners, however, the lack of referral between the two can be attributed to poor healthcare regulations that don't fully recognize traditional health practices. The South African Health system does not currently address issues of referring patients outside the boundaries of the allopathic healthcare system, therefore such referrals to traditional healthcare practices would be a challenging exercise.

Although there were no formal ways of referring patients in this study, differing methods of ensuring patients are referred could be applied. Traditional health practitioners are usually vested in their patient care; therefore, they have an obligation to address and assess their patients and ensure that treatment is received. Furthermore, they share the patient's culture and beliefs about diseases and have a holistic approach to patient care^17^.

Allopathic healthcare systems make use of referral letters to refer patients from one service to another and that assists with record keeping and ensuring continuity of care. The referral from an allopathic healthcare practitioner's point of view would be a letter with a detailed summary of patient presentation, diagnostic processes, and current management together with an indication of why further clinical management is sought^18^. The lack of regulated referral routes between the two systems lead to traditional health practitioners verbally referring patients and no referral records were kept. The lack of formal communication makes the verbal referral done to be more of health advice than a referral.

Other studies found practices in referral indicating that traditional healthcare practitioners usually delayed patient referral until it is too late^11^. However, in this study, the traditional health practitioners cited an almost immediate referral depending on the patient's condition. Patients are not touched until the medical aspect of the illness is catered for at the clinic or hospital. This is because traditional healthcare practitioners care for the spiritual being and the physical being taking into consideration the patient's environment as an influence to their health. This study did not test possible treatment failure as a reason for referral, therefore that could not be concluded, however, participants noted their limitations in case of patient care and this was a primary reason for patient referral.

Referral from traditional healthcare practitioners to allopathic healthcare is sometimes a temporary measure^19^. Hence in this study, it is usually the case that patients are referred and informed to return once care from the allopathic healthcare practitioner had been initiated or completed, same was a finding in this study. This is because typically the kind of healthcare sought from the traditional healthcare practitioner often differ with that of the allopathic. They address differing needs from spiritual and physical needs.

Allopathic healthcare practitioners do not acknowledge referral from traditional healthcare practitioners or give feedback regarding patients' progress. As such, it was not known how the patients are doing until they have returned from the allopathic healthcare practices with feedback to the traditional healthcare practitioners at their next consultation.

### Implications for practice

The results from this study indicate a strong need for an understanding of the South African indigenous knowledge systems and the traditional health practices. People's beliefs will direct their health-seeking behavior and that is where parallel use of both traditional and allopathic healthcare systems is seen. Understanding the traditional healthcare practices sought by patients could advance understanding of the referral systems to promote a collaborative effort between the two systems of healthcare and improve continuity of care.

## Limitations

Limitations were experienced in this study and they included study setting. The study was done focusing on practitioners in that township with reference to their clinic facilities. The results may not be transferrable to another context as participants from another location may have differing views supported by their experience. Another limitation experienced is the dearth of previous studies relating to traditional healthcare practitioner's referral practices to support findings.

## Conclusion

The patient's referral system in South Africa is a fruitful and functional system between different levels of allopathic healthcare. However, much still needs to be done to advance collaboration between the two systems of healthcare that currently exists as separate entities. Patients as cultural beings are users of both system and in all interactions, patient care remains a priority. There is still a need to conduct more studies around traditional healthcare practices in order to address some of the challenges faced by patients and healthcare practitioners.
